# Comparing Macular Thickness Measurements in Patients with Diabetic Macular Edema with the Optos Spectral OCT/SLO and Heidelberg Spectralis HRA + OCT

**DOI:** 10.3390/vision1010002

**Published:** 2016-04-12

**Authors:** Amun Sachdev, Magdalena Edington, Rupal Morjaria, Ngaihang Victor Chong

**Affiliations:** 1 Oxford Eye Hospital, University of Oxford, Oxford, Oxfordshire, OX3 9DU, UK; 2 Sandwell & West Birmingham Hospitals NHS Trust, Dudley road, Birmingham, West Midlands, B18 7QH, UK; 3 NHS Greater Glasgow and Clyde, 1055 Great Western Road, Glasgow, G12 0YN, UK

**Keywords:** diabetic macular edema, optical coherence tomography, retinal thickness, heidelberg spectralis, optos spectral OCT/SLO

## Abstract

The aim of this study was to compare measurements of macular thickness, obtained from patients with diabetic macular edema, using two spectral-domain optical coherence tomography (SD-OCT) devices. These were the Spectralis Heidelberg Retina Angiograph + Optical Coherence Tomography (HRA + OCT) (Heidelberg Engineering), which is often considered the gold-standard for OCT measurement, and the Spectral Optical Coherence Tomography/Scanning Laser Ophthalmoscopy (OCT/SLO) (Optos plc), which can additionally perform microperimetry, a useful measure of visual function. In this prospective observational study, each eye had SD-OCT performed with both devices on the same day by the same investigator. Mean retinal thickness was calculated, and compared between the devices, for central and parafoveal zones within 3 mm of the fovea. 62 eyes were included. In the central, superior, temporal, inferior and nasal zones respectively, mean retinal thickness with Spectralis HRA+OCT was (in microns) 310, 343, 344, 332 and 340; measurements with Spectral OCT/SLO were 237, 298, 297, 289 and 290. Pearson correlations between the devices were 0.752, 0.85, 0.928, 0.839, and 0.823 (*p* < 0.0001). Although absolute measurements between the devices were significantly different and therefore not interchangeable, the correlation between the devices was over 75% and statistically significant in all zones. Thus, the Spectral OCT/SLO could reliably be used for SD-OCT in patients who may also require microperimetry assessment.

## 1. Introduction

Diabetic retinopathy is the most common microvascular complication of diabetes [1] and the 10-year incidence of diabetic macular edema (DME) is 14% [2]. Destruction of the blood–retinal barrier due to loss of pericytes and endothelial cell junctions causes leakage of plasma fluid from compromised macular blood vessels, producing macular oedema and thickening. This results in a decline in visual acuity and contrast sensitivity, particularly with macular oedema involving the fovea [3].

New imaging techniques such as optical coherence tomography (OCT) imaging and more recently microperimetry, have allowed improved assessment and treatment of DME. OCT was first introduced in 1991 [4] allowing accurate, high-resolution, non-invasive, *in vivo* visualisation of the internal cross-sectional microstructure of the retina. OCT images were first acquired in a time-domain fashion (TD-OCT) in which a light beam is focussed upon the retina and the reflected light is recombined from a reference and a sample arm in order to determine depth. Spectral Domain Optical Coherent Tomography (SD-OCT) was subsequently introduced, allowing an increased scan rate and number, reducing the effect of motion artefact and enhancing resolution. In the medical retina field, it is often relied upon for the early detection and staging of DME, as well as monitoring anatomical changes that can occur with disease progression and in response to therapy [5]. However, from the patient’s point of view, the functional approach to retinal diseases such as DME is more relevant than a morphological approach. Studies have demonstrated only a modest correlation between retinal thickness and visual acuity [6]. This has led to the increasing use of microperimetry. 

Microperimetry has been used to evaluate retinal function and thus reflect the impact of vision upon quality of life more accurately than visual acuity [7,8,9,10,11,12,13,14,15]. This allows exact topographical correlation between retinal thickness and light sensitivity by projecting a defined light stimulus over a selected location in real-time, which is independent of eye movements or fixation. Microperimetry has already been used to successfully determine the relationship between retinal sensitivity and structural changes detected with SD-OCT in DME [7,8,9], branch retinal vein occlusion (BRVO) [10], geographic atrophy [11] and glaucoma [12], as well as monitoring responses to treatments in DME [13], BRVO [14] and geographic atrophy [15]. As such, microperimetry is valuable tool which can be used in clinical practice to provide further information regarding a patient’s functional vision and monitor this in conditions such as DME. 

Whilst the Spectralis Heidelberg Retina Angiograph + Optical Coherence Tomography (HRA + OCT) is often considered the gold standard of SD-OCT, it does not have an integrated microperimetry module and is therefore unable to measure retinal sensitivity. However, the Optos Spectral Optical Coherence Tomography/Scanning Laser Ophthalmoscopy (OCT/SLO) has both SD-OCT and microperimetry modules integrated in the same device, allowing accurate and reliable point-to-point co-localisation. Previous studies have demonstrated that this device has good reproducibility for macular retinal thickness measurements [16,17].

Various studies have sought to compare retinal thickness measurements between different OCT machines in healthy subjects [16,17,18,19,20]. In patients with DME, the Heidelberg Spectralis HRA + OCT has been compared with other SD-OCT devices such as the Cirrus SD-OCT (Carl Zeiss Meditec, Inc., Dublin, CA, USA) and TD-OCT devices such as the Stratus OCT (Carl Zeiss Meditec, Inc., Dublin, CA, USA) [17,21,22,23,24,25]. Other studies have only measured the central retinal thickness in the 1 mm area around the fovea and did not include the parafoveal zones in their analysis [16,19,22]. 

In our study, we determine the clinical application of two SD-OCT devices that have not previously been compared in patients with DME, by studying whether there is a correlation between them. We compare an SD-OCT device often seen as the gold-standard for OCT measurement (Heidelberg Spectralis HRA + OCT) to another SD-OCT device which can also perform microperimetry (Optos Spectral OCT/SLO), an investigation which has been shown to be a useful measure of visual function [7,8,9,10,11,12,13,14,15] and may therefore have clinical application in retinal diseases such as DME. We include both the central and parafoveal zones in our analysis, encompassing a total diameter of 3 mm around the fovea. This provides a larger surface area, thus increasing the likelihood that it will contain diabetic macular pathology, which can significantly impact upon a patient’s visual function.

This is the first study to report a comparison of retinal thickness measurements obtained from the Heidelberg Spectralis HRA + OCT and the Optos Spectral OCT/SLO in patients with DME. In this way, we aim to determine whether the Optos Spectral OCT/SLO might be used as a feasible alternative for patients with pathology that requires both SD-OCT and microperimetry. Indeed, we found that whilst absolute measurements of macular thickness were significantly different between the two devices, and therefore not interchangeable, there is a good correlation between the measurements. As such, the Optos Spectral OCT/SLO could reliably be used in patients who require both SD-OCT measurements and microperimetry assessment. 

## 2. Results

Sixty-two eyes of 50 patients (38 male, 12 female) with DME were included in the study. Of these patients, 12 provided measurements from both eyes. The mean age was 58.1 years (range 30–85) and the mean BCVA was 77.2 letters on ETDRS (range 55–92).

The mean retinal thickness, as measured by Heidelberg Spectralis HRA + OCT and Optos Spectral OCT/SLO, in the central, superior, temporal, inferior and nasal zones are shown in Table 1. 

Compared to the Optos Spectral OCT/SLO, the Heidelberg Spectralis HRA + OCT demonstrated higher mean retinal thickness measurements in all five zones. Between the measurements from the devices, there was a difference of 73 µm in the central zone and 43–50 µm in the parafoveal zones This difference between these absolute measurements was highly significant in all zones (*p* < 1 × 10^−7^). The Pearson Correlation was 0.752 in the central zone, and greater than 0.820 in the parafoveal zones. This was also highly significant in all zones (*p* < 0.0001).

## 3. Discussion

SD-OCT has become widely accepted in detecting and monitoring a variety of retinal diseases in clinical ophthalmological practice [5] and microperimetry is becoming increasingly recognised as a useful tool in measuring retinal sensitivity DME [7]. As such, it is becoming increasingly important to explore the validity of devices, such as the Optos Spectral OCT/SLO, which integrate these two features and thus allow an assessment of both the macular anatomy and corresponding function.

In this study, we compared the central and parafoveal retinal thickness measurements from the Optos Spectral OCT/SLO and Heidelberg Spectralis HRA + OCT in patients with DME. We observed that the correlation between the mean retinal thickness measurements taken with the two devices was over 75% in all zones. This suggests that the measurements achieved from the Optos Spectral OCT/SLO are consistent with those from the Heidelberg Spectralis HRA + OCT, often considered to be the gold-standard for SD-OCT. However, the absolute measurements from the two devices were significantly different in all zones, suggesting that measurements would not be interchangeable between the devices. In all the individual zones, the Heidelberg Spectralis HRA + OCT yielded higher measurements for mean retinal thickness.

There have been previous studies that have recruited patients with DME, but used other SD-OCT or TD-OCT devices to compare retinal thickness measurements [23,24,25]. Similar to our study, Lammer J. *et al.* [23] found that there was a significant difference and correlation between the measurements from different OCT devices.

**Figure 1:**
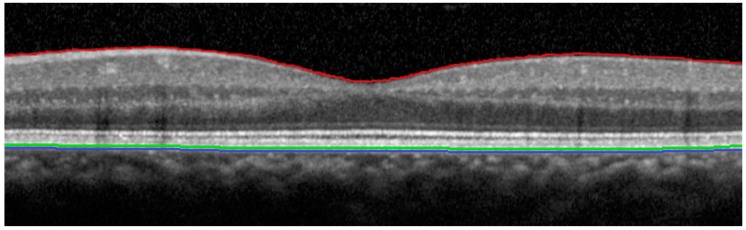
OCT scan from a healthy subject, demonstrating the different retinal layers that are used to define the outer retinal border for each SD-OCT device. Red line: Vitreo-retinal interface. Green line: Outermost retinal border defined by the Spectral OCT/ SLO (Optos plc, Dunfermline, Scotland, UK). Blue line: Outermost retinal border defined by the Spectralis HRA + OCT (Heidelberg Engineering, Heidelberg, Germany).

Two previously published studies that compared macular retinal thickness measurements between the Optos Spectral OCT/SLO and Heidelberg Spectralis HRA + OCT [16,18] did not analyse the correlation between the two devices. They were both conducted in healthy subjects and Wolf-Schnurrbusch *et al*. [16] only analysed the central retinal thickness in the 1 mm area around the fovea; however, similar to our study, they also reported higher retinal thickness measurements with the Heidelberg Spectralis HRA + OCT compared to the Optos Spectral OCT/SLO. Whilst their central retinal thickness measurements revealed a 45 µm [16] and 57–60 µm [18] difference between the devices, our study found a difference of 73 µm in the central zone and 43–50 µm in the parafoveal zones. These studies [16,18] have proposed that as both devices use different software for segmentation of the outer retinal border, with the Spectralis HRA + OCT identifying a deeper reflective border as the outermost retina, then this result is not entirely unexpected; whilst the Heidelberg Spectralis HRA + OCT measures retinal thickness between the vitreo-retinal interface and the outermost reflective band of the RPE, the Spectral OCT/SLO measures retinal thickness to the mid-RPE reflectance instead (Figure 1). Other studies have also proposed this as a cause for the differences between macular thickness measurements when comparing other OCT devices [17,19,21,22,24,25]. However, there may also be a variety of other factors influencing macular thickness measurements between the devices, in addition to differences in defining segmentation algorithms. For example, devices may also differ in their scan patterns and methods of signal collection [16,18,19,22,25], tracking and axial resolution [18,19,25], optics [16], and imaging processing software algorithms [16,18,19,25]. Unlike Wolf-Schnurrbusch *et al*. [16] and other studies [19,22], we measured the retinal thickness for the central circle and four parafoveal zones, encompassing the central 3.0 mm around the fovea. This is because the normal anatomy of the retina involves a gradual increase in thickness as the distance from the fovea increases; as such, a single foveal measurement would depend more greatly upon accurate centration than the totality of an area of 3.0 mm around the fovea.

Limitations of our study included the relatively small number of patients participating in the study. In addition, we have only evaluated two SD-OCT devices. Further studies would be required to determine whether our observations are also found in other SD-OCT devices in patients with DME.

In summary, this study has provided additional information on the reliability of using the Optos Spectral OCT/SLO in patients with macular pathology, namely DME. Indeed, our findings propose that whilst it is not possible to transfer absolute measurements between the devices, there is a very good correlation between the measurements from both devices, the Heidelberg Spectralis HRA + OCT and the Optos Spectral OCT/SLO. This suggests that the Optos Spectral OCT/SLO could reliably be used for SD-OCT in patients who also require microperimetry assessment, as long as the patient continues to be monitored with this same device during the management of their condition.

## 4. Materials and Methods

This was a prospective cross-sectional observational study. Consecutive patients were recruited from diabetic retinopathy clinics, over a period of 6 months from February 2012 to July 2012, after meeting the following inclusion criteria: age 18 or over; best corrected visual acuity on Snellen chart of 20/80 or better; no history of any other previous ocular disease beyond refractive error and cataract extraction. All recruited patients had a confirmed diagnosis of DME made by a consultant ophthalmologist (Ngaihang Victor Chong) with experience in managing patients with diabetic retinopathy. This research adhered to the tenets of the Declaration of Helsinki and all subjects gave their informed consent.

### 4.1. Data Collection

Data was collected by selected trained investigators (Amun Sachdev or Magdalena Edington). Before OCT examination, best corrected visual acuity (BCVA) was measured in all patients with both the Snellen chart and letter counting with the ETDRS (Early Treatment Diabetic Retinopathy Study) visual acuity chart. The pupils of the study eyes were subsequently dilated with drops containing 1% tropicamide and 2.5% phenylephrine. SD-OCT was then performed with both the Spectralis HRA + OCT (Heidelberg Engineering, Heidelberg, Germany) and the Optos Spectral OCT/SLO (Optos plc, Dunfermline, Scotland, UK). For each eye, all SD-OCT measurements were performed on the same day by the same investigator with both devices.

The Spectral OCT/SLO uses a combination of OCT and confocal scanning laser ophthalmoscopy (SLO) to produce a 3D retinal topography map, with a 512 × 64 scan pattern, covering an area of 9.0 mm × 9.0 mm. The Heidelberg Spectralis HRA + OCT scanning protocol was the “high speed mode” with 768-A scans/B-scan, and 19 B-scans obtained over an area of 30 × 15 degrees, averaging 9 scans per B-scan. Both devices incorporate an integrated eye tracking system which compensates for eye movement artefact, thus improving the reproducibility of foveal centre selection during data acquisition.

**Figure 2:**
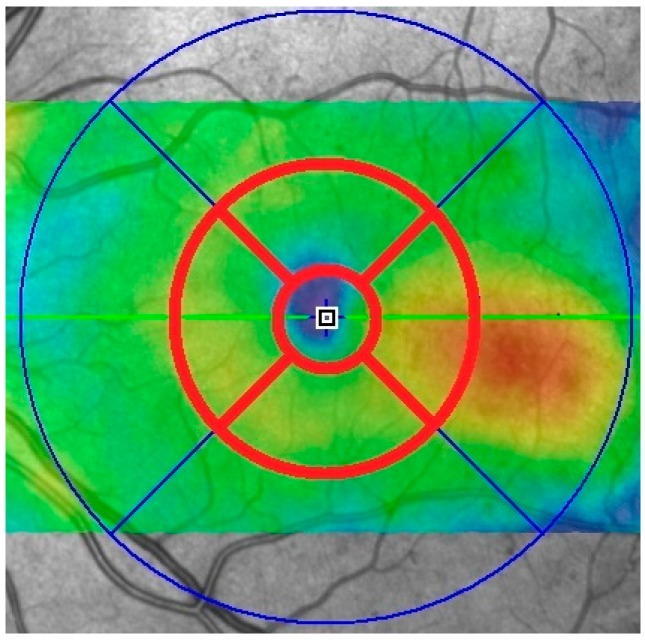
Scan for a patient using Spectralis HRA + OCT (Heidelberg Engineering, Heidelberg, Germany). The central and parafoveal zones that were used during data analysis are highlighted.

**Figure 3:**
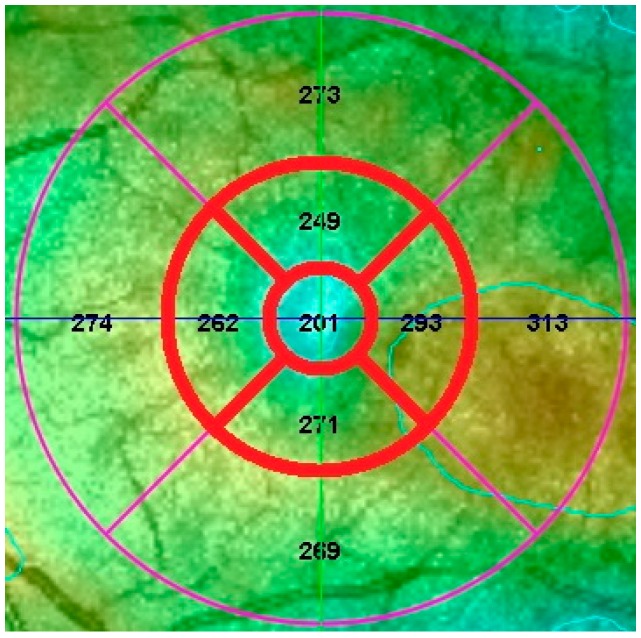
Scan for a patient using Spectral OCT/SLO (Optos plc, Dunfermline, Scotland, UK). The central and parafoveal zones that were used during data analysis are highlighted. The mean retinal thickness for each zone is shown.

The software analysis for both devices involved measuring the mean retinal thickness through nine different zones: the central circle (1.0 mm in diameter), an inner ring divided into four quadrants (covering the diameter between 1.0 and 3.0 mm and the fovea) and an outer ring also divided into four quadrants (covering the diameter between 3.0 and 6.0 mm and the fovea) (Figure 2 and Figure 3).

### 4.2. Data Analysis

Images from both devices were reviewed for each patient in order to ensure that they were centralised appropriately for measurement of retinal thickness. They were reviewed independently by two investigators (Amun Sachdev and Magdalena Edington) before data analysis. If there was any variability, the senior researcher (Ngaihang Victor Chong) would review the images. Investigated eyes were excluded from the study if the images gained from one or both devices were too poor in quality to gain reliable data (for example, due to imaging artefacts or segmentation errors).

The retinal thickness was determined for the central circle and the four parafoveal zones of the inner ring for both devices (Figure 2 and Figure 3), measuring the central 3.0 mm around the fovea. For the Heidelberg Spectralis HRA + OCT, retinal thickness was the distance between the vitreo-retinal interface and the outermost reflective band of the retinal pigment epithelium (RPE). For the Spectral OCT/SLO, retinal thickness was defined as the distance between the vitreo-retinal interface and the mid-RPE reflectance (Figure 1).

For both the Heidelberg Spectralis HRA + OCT and the Optos Spectral OCT/SLO, the mean retinal thickness of the entire patient cohort was calculated for each zone. Correlation between the mean retinal thickness measurements from the two devices was determined using student’s paired *t*-test. *p* < 0.05 was considered statistically significant. Pearson correlation was also calculated to assess the relationship between the two devices. Statistical analysis was performed using SPSS software (version 22.0; SPSS Inc., Chicago, IL, USA).

## Figures and Tables

**Table 1:** Comparison of the mean retinal thickness from both spectral-domain optical coherence tomography (SD-OCT) devices. The mean retinal thickness (in microns) in the five zones lying within 3 mm of the central fovea, as measured by Spectralis Heidelberg Retina Angiograph + Optical Coherence Tomography (HRA + OCT) (Heidelberg Engineering, Heidelberg, Germany) and Spectral Optical Coherence Tomography/Scanning Laser Ophthalmoscopy (OCT/SLO) (Optos plc, Scotland).

Devices/Statistical Comparison	Central Zone	Superior Zone	Temporal Zone	Inferior Zone	Nasal Zone
Heidelberg Spectralis HRA + OCT	310	343	344	332	340
Optos Spectral OCT/SLO	237	298	297	289	290
Absolute difference	73	45	47	43	50
*p*-value	1.96 × 10^−14^	5.46 × 10^−1^	3.51 × 10^−8^	1.63 × 10^−13^	2.85 × 10^−12^
Pearson Correlation	0.752	0.85	0.928	0.839	0.823

## Abbreviations

The following abbreviations are used in this manuscript:

OCT: Optical coherence tomography
HRA: Heidelberg Retina Angiograph
SD-OCT: Spectral-domain optical coherence tomography
TD-OCT: Time-domain optical coherence tomography
SLO: Scanning laser ophthalmoscopy
DME: Diabetic macular edema
BRVO: Branch retinal vein occlusion
BCVA: Best corrected visual acuity
ETDRS: Early treatment diabetic retinopathy study
RPE: Retinal pigment epithelium
